# Testing for individual differences in the effects of men’s physical attractiveness and perceived abusiveness on women’s hypothetical dating decisions

**DOI:** 10.1038/s41598-025-07575-5

**Published:** 2025-07-01

**Authors:** Kathlyne Leger, Benedict C. Jones, Victor K. M. Shiramizu

**Affiliations:** School of Psychological Sciences & Health, University of Strathclyde, Glasgow, UK

**Keywords:** Psychology, Human behaviour

## Abstract

Romantic-partner choice is a fundamental human behaviour. However, the factors that influence partner choice remain poorly understood. Here we investigated (1) how women’s first impressions of potential partners’ physical attractiveness and potential for abusive behaviour based on face images influence hypothetical dating decisions and (2) possible moderating effects of individual differences in women’s sensation seeking, sociosexual orientation (i.e., openness to uncommitted sexual relationships), current partnership status, and self-perceived mate value (i.e., self-rated attractiveness). Physical attractiveness of potential dates, but not perceptions of potential for abusive behaviour, was a strong predictor of reported dating intentions, but this effect of physical attractiveness was weaker among women who scored higher on sensation seeking. None of the other individual-difference variables had significant effects. Collectively, these findings present further evidence for the importance of physical attractiveness in dating and partner choices and highlight the role sensation seeking appears to play in individual differences in the effect of men’s physical attractiveness on women’s dating intentions.

## Introduction

Although choosing a romantic partner is a fundamental human behaviour, the factors that influence romantic-partner choice remain poorly understood. Nonetheless, multiple lines of evidence suggest that the physical attractiveness of potential partners plays a key role in romantic-partner choices. For example, people prefer to date and marry more physically attractive individuals (see^1^ for a meta-analytic review) and the physical attractiveness of potential partners is a good predictor of both swiping decisions made on dating apps^2^ and dating decisions made following speed-dating events^3^.

People form a wide range of first impressions of individuals’ personality traits based on facial appearance (see^4–6^ for reviews) and it is well-established that these first impressions, though often inaccurate, can influence subsequent behaviour (see^5,6^ for a review). Consequently, Olivera-La Rosa et al.^7^ recently proposed that a better understanding of the role these first impressions play in dating decisions might provide important insights into the processes that influence such decisions, particularly in the context of choices made on dating apps. Given that dating increases women’s risk of violence (and sexual violence in particular^8^), one might reasonably expect that first impressions of faces relating to potential partners’ propensity to abuse their partners would play an important role in women’s dating decisions. Indeed, Shuster et al.^9^ recently reported that threat-related perceptions of potential partners’ face images had a negative effect on reported dating intentions in a hypothetical dating-decision task.

In light of the above, the current study investigated the roles that attractiveness- and violence-related perceptions play in hypothetical dating decisions when heterosexual women assessed images of men’s faces. Male faces were first rated by one group of women for a range of attractiveness- and violence-related traits. These ratings were then subjected to Principal Component Analysis (PCA) to identify the underlying dimensions. This PCA showed that the trait ratings were underpinned by distinct physical attractiveness and perceived abusiveness dimensions. Next, a different group of women assessed the same male faces in a hypothetical dating decisions task where they indicated how likely they would be to date each man. Because some previous research has reported that people are often more willing to discount negative traits (e.g., a sexual history suggesting an individual may be more likely to have sexually transmitted infections) when assessing more physically attractive potential partners^10,11^, the physical attractiveness and perceived abusiveness dimensions may interact to predict hypothetical dating decisions.

In addition to the above, we tested for possible moderating effects of individual differences in women’s sensation seeking, sociosexual orientation (i.e., openness to uncommitted sexual relationships), current partnership status, and self-perceived mate value (i.e., self-rated attractiveness). Sociosexual orientation, current partnership status, and self-perceived mate value were assessed because of previous research suggesting that women who consider themselves to be more attractive (see^12^ for a review), are currently in a relationship (see^13^ for a review), or who are more open to uncommitted sexual relationships (see^14^ for a review) show stronger preferences for masculine and/or physically attractive men. Sensation seeking was assessed because of previous work suggesting that sensation seeking predicts individual differences in how people integrate traits that are desirable and undesirable traits in potential partners^11^.

## Methods

### Ethics

All procedures were approved by the Department of Psychological Sciences and Health (University of Strathclyde) Ethics Committee, all work was undertaken in accordance with the Declaration of Helsinki, and all participants provided informed consent.

### Stimuli

**Fig. 1:**
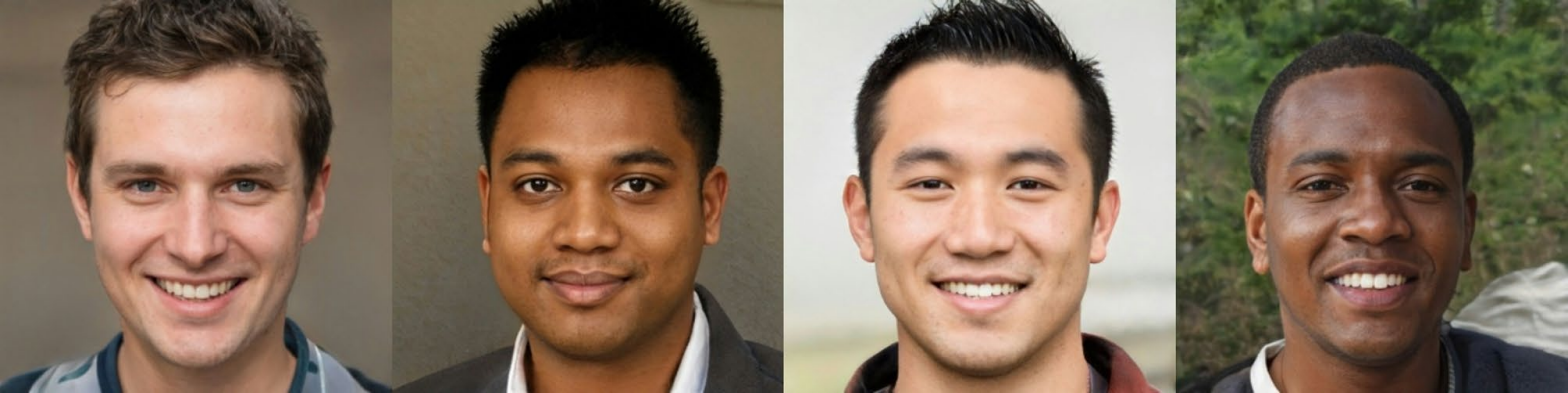
Examples of face stimuli used in our study.

Stimuli were taken from an open-access database^15^ of 200 AI-generated male faces of diverse ethnicity (Black, White, East Asian, and West Asian faces) shown mostly with smiling expressions (see Fig. 1 for example images). Nightingale and Farid^15^ have previously shown that people cannot distinguish these AI-generated images from control images (i.e., images of real people). Twenty heterosexual women (mean age = 29.75 years, SD = 3.58 years) were recruited via prolific and judged the age of these 200 male faces (mean age = 32.25 years, SD = 14.06 years, Cronbach’s alpha for age judgments = 0.98). Trial order was fully randomised. These age ratings were used to randomly select 25 Black, 25 White, 25 East Asian, and 25 West Asian faces that were perceived, on average, to be between 18 and 40 years of age (mean age of this subset of images = 29.45 years, SD = 5.29 years). These 100 male faces images served as stimuli for the subsequent parts of the study. All data and analysis code for this part of our study are publicly available on the Open Science Framework (https://osf.io/ugpxs/).

### Deriving perceived abusiveness and physical attractiveness components

Two hundred and thirteen heterosexual women (mean age = 28.89 years, SD = 4.23 years) were recruited via Prolific and randomly allocated to rate the 100 male faces for one of eight questions using a seven-point scale (1 = much less than average, 7 = much more than average). The eight questions were “How likely is this man to resolve arguments with their partner or a potential partner by discussing the problems?” (Cronbach’s alpha = 0.87), “How likely is this man to behave aggressively towards their partner or a potential partner by shouting, swearing at them, or insulting them?” (Cronbach’s alpha = 0.81), “How likely is this man to physically assault their partner or a potential partner?” (Cronbach’s alpha = 0.86), “How likely is this man to coerce their partner or a potential partner to have sex with them?” (Cronbach’s alpha = 0.81), “How likely is this man to physically injure their partner or a potential partner?” (Cronbach’s alpha = 0.85), “How attractive is this man?” (Cronbach’s alpha = 0.89), “How good looking is this man?” (Cronbach’s alpha = 0.90), and “How sexy is this man?” (Cronbach’s alpha = 0.92). The first five questions described above were chosen to reflect the five subscales of Straus et al.’s^16^ Revised Conflict Tactics Scale (negotiation, psychological aggression, physical assault, sexual coercion, injury), which assesses both the extent to which partners engage in physical and psychological attacks and the extent to which partners use negotiation and reasoning to deal with conflict. Trial order was fully randomised.

Next, we calculated the mean rating for each face on each of these traits and subjected these scores to Principal Component Analysis (PCA) with varimax rotation using the R package Psych 2.3.6^17^. This PCA produced two components that explained 49% and 41% of the variance in scores, respectively. The first component was highly correlated with ratings for the five questions assessing perceptions of the likelihood of engaging in abusive behaviours. We labelled this component the Perceived Abusiveness component. The second component was highly correlated with ratings for the three questions assessing perceptions of physical attractiveness. We labelled this component the Physical Attractiveness component. Correlations between individual traits and both components are shown in Table 1. All data (i.e., ratings) and analysis code for this part of our study are publicly available on the Open Science Framework (https://osf.io/ugpxs/). Images that scored highest and lowest on each component are also available on the Open Science Framework (https://osf.io/ugpxs/).

**Table 1:** Correlations between individual traits and the perceived abusiveness and physical attractiveness components.

	Perceived abusiveness component	Physical attractiveness component
How likely is this man to physically assault their partner or a potential partner?	**0.945**	0.012
How likely is this man to behave aggressively towards their partner or a potential partner by shouting, swearing at them, or insulting them?	**0.941**	0.070
How likely is this man to physically injure their partner or a potential partner?	**0.932**	0.176
How likely is this man to coerce their partner or a potential partner to have sex with them?	**0.743**	0.574
How sexy is this man?	0.116	**0.963**
How good looking is this man?	−0.006	**0.967**
How attractive is this man?	−0.027	**0.965**
How likely is this man to resolve arguments with their partner or a potential partner by discussing the problems?	**−0.850**	0.393

Correlations with absolute values greater than 0.7 are shown in bold.

### Hypothetical dating-decision task and individual-difference measures

One hundred heterosexual women (mean age = 28.39 years, SD = 4.37 years) were recruited from Prolific and rated the 100 male faces for the question “If you were looking for someone to date, how interested would you be in dating this person?” using a seven-point scale (1 = not very, 7 = very). Trial order was fully randomised.

These women also completed Penke and Asendorpf’s^18^ Revised Sociosexual Orientation Inventory (SOI-R, Cronbach’s alpha = 0.80, M = 27.11), Hoyle et al.’s^19^ Brief Sensation Seeking Scale (BSSS, Cronbach’s alpha = 0.82, M = 21.21), and Edlund and Sagarin’s^20^ Mate Value Scale (MVS, Cronbach’s alpha = 0.93, M = 17.99). Participants also reported whether they were currently in a romantic relationship (65 women were in a relationship, 35 women were not in a relationship).

Penke and Asendorpf’s^18^ Revised Sociosexual Orientation Inventory is a nine-item questionnaire measuring openness to uncommitted sexual relationships. Participants respond to questions such as “With how many different partners have you had sexual intercourse without having an interest in a long-term committed relationship with this person?”, using a nine-point scale. Hoyle et al.’s^19^ Brief Sensation Seeking Scale is an eight-item questionnaire measuring dispositional sensation seeking. Participants respond to questions such as “I like wild parties” using a five-point scale. Edlund and Sagarin’s^20^ Mate Value Scale is a four-item questionnaire assessing perceptions of own mate value. Participants respond to questions such as “Overall, how would you rate your level of desirability as a partner on the following scale?”, using a seven-point scale. For each questionnaire, a single score can be calculated, with higher scores indicating greater openness to uncommitted sexual relationships, higher sensation seeking, and higher mate value, respectively. The order in which participants completed the hypothetical dating-decision task and questionnaires was fully randomised. Scores on the BSSS were positively and significantly correlated with scores on the SOI-R (*r* = .32, *N* = 100, *p* = .001) and positively correlated with scores on the MVS, although this correlation was not significant (*r* = .19, *N* = 100, *p* = .056). Scores on the MVS and SOI-R were not significantly correlated (*r* = .12, *N* = 100, *p* = .121).

## Results

All data and analysis code are publicly available on the Open Science Framework (https://osf.io/ugpxs/). Responses were analysed using a linear mixed effects model carried out in RStudio^21^ using the lmerTest 3.1.3^22^ package. Responses on the hypothetical dating-decision task served as our dependent variable. Predictors were scores on the Perceived Abusiveness component, Physical Attractiveness component, SOI-R (z-scored), MVS (z-scored), BSSS (z-scored), and partnership status (effect coded so that 0.5 corresponded to being in a relationship and − 0.5 corresponded to not being in a relationship). The model included all possible two-way and three-way interactions, except for those involving multiple individual-difference measures (i.e., possible interactions including more than one of SOI-R, MVS, BSSS, or partnership status were not included in the model). The model also included, by-rater and by-stimulus random intercepts, by-rater random slopes for the interaction between Perceived Abusiveness component and Physical Attractiveness component, and by-stimulus random slopes for SOI-R (z-scored), MVS (z-scored), BSSS (z-scored), and partnership status. Full results of this analysis are shown in Table 2.

**Table 2:** Results of our linear mixed effects model.

	Estimate	SE	t	df	*p*
Intercept	2.473	0.102	24.275	102.746	< 0.001
Perceived abusiveness component	−0.013	0.023	−0.563	136.526	0.574
Physical attractiveness component	0.600	0.039	15.589	124.76	< 0.001
SOI-R	0.102	0.104	0.979	99.140	0.330
MVS	−0.144	0.105	−1.376	99.257	0.172
BSSS	−0.198	0.103	−1.910	99.876	0.059
Partnership status	0.118	0.207	0.570	99.048	0.570
Perceived abusiveness component * Physical attractiveness component	0.011	0.019	0.569	106.956	0.570
Perceived abusiveness component * SOI-R	0.016	0.019	0.861	92.871	0.391
Physical attractiveness component * SOI-R	0.021	0.037	0.557	99.458	0.579
Perceived abusiveness component * MVS	0.012	0.019	0.614	94.400	0.540
Physical attractiveness component * MVS	-0.017	0.037	-0.459	100.266	0.647
Perceived abusiveness component * BSSS	0.025	0.020	1.288	107.029	0.201
Physical attractiveness component * BSSS	−0.077	0.037	−2.055	104.888	0.042
Perceived abusiveness component * Partnership status	0.020	0.037	0.546	90.524	0.587
Physical attractiveness component * Partnership status	0.100	0.074	1.363	98.752	0.176
Perceived abusiveness component * Physical attractiveness component * SOI-R	−0.009	0.013	−0.692	72.663	0.491
Perceived abusiveness component * Physical attractiveness component * MVS	0.011	0.013	0.823	73.398	0.413
Perceived abusiveness component * Physical attractiveness component * BSSS	0.017	0.014	1.171	87.984	0.245
Perceived abusiveness component * Physical attractiveness component * Partnership status	−0.006	0.026	−0.240	69.261	0.811

**Fig. 2:**
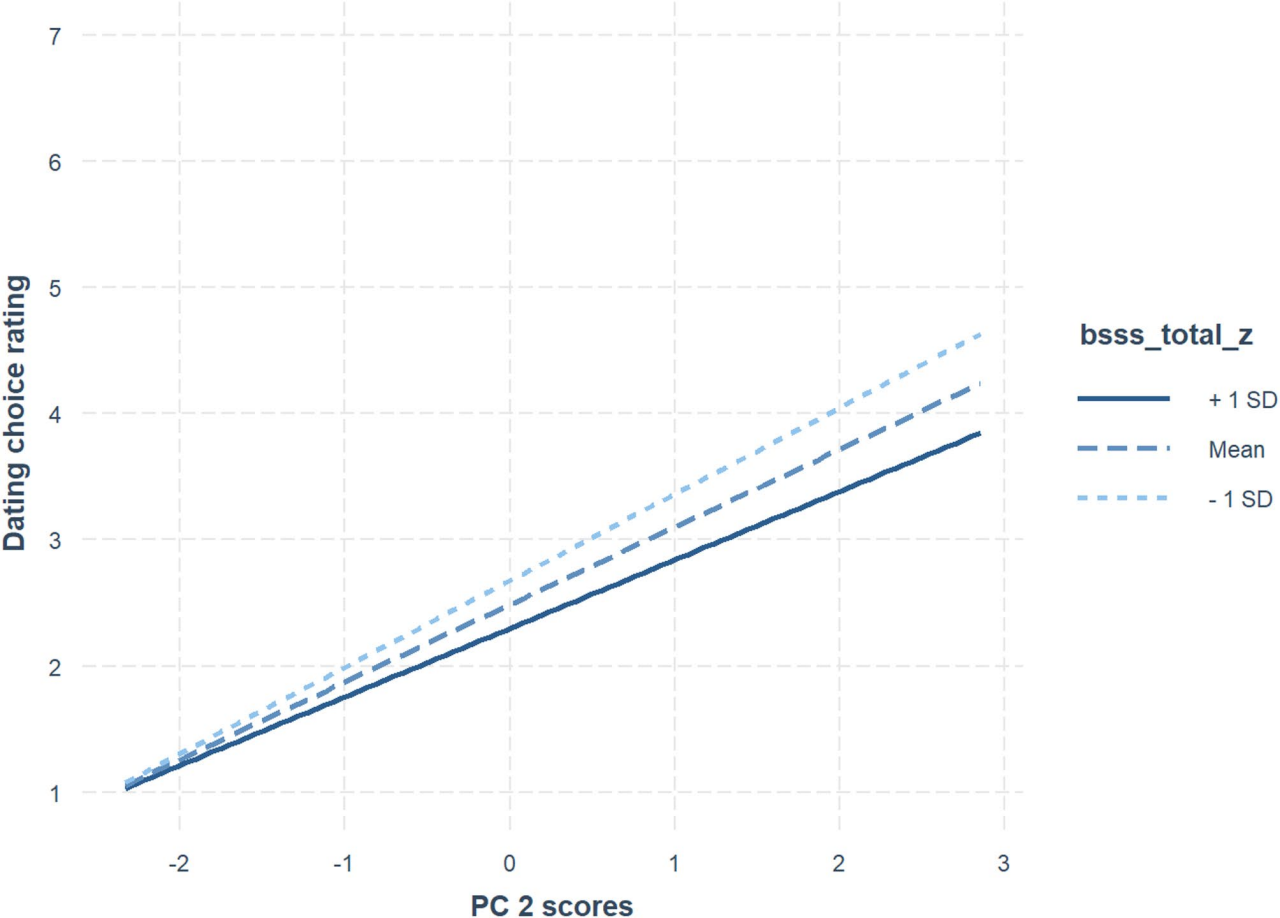
The significant interaction between the physical attractiveness component (PC2) and BSSS (brief sensation seeking scale).

There was a significant positive effect of the Physical Attractiveness component (estimate = 0.600, SE = 0.039, t = 15.589, df = 124.760, *p* < .001) and a significant interaction between the Physical Attractiveness component and BSSS (estimate = -0.077, SE = 0.037, t = -2.055, df = 104.888, *p* = .042). This interaction is shown in Fig. 2. No other effects were significant. Pseudo R squares for this model were 0.166 (fixed effects) and 0.599 (total). Simple slopes analyses showed that the interaction between the Physical Attractiveness component and BSSS occurred because women who scored higher on the BSSS showed a weaker effect of the Physical Attractiveness component on hypothetical dating decisions (see https://osf.io/ugpxs/ for full results of this simple slopes analysis).

At the suggestion of a Reviewer, we ran an additional version of our main analysis, this time excluding women who responded 1 on 75% or more of the trials as a robustness check. This analysis excluded 28 women and showed the same pattern of results, although here the interaction between physical attractiveness and BSSS was not significant (*p* = .056). This robustness check is reported in full at https://osf.io/ugpxs/.

We also carried out simplified versions of our main analysis, in which we tested for possible effects of SOI-R, MVS, BSSS, and partnership status in separate models (i.e., we ran four further models in total, each with a different individual differences measure included in the model). The effect of the Physical Attractiveness component was significant and positive in all four models (all estimates > 0.605, all t > 15.526, all *p* < .001). Neither the effect of the Perceived Abusiveness component nor the interaction between the Physical Attractiveness and Perceived Abusiveness components was significant in any of these models. In these models, the only significant effect that involved any of our individual difference measures (SOI-R, MVS, BSSS, and partnership status) was a significant interaction between scores on the Physical Attractiveness component and scores on the BSSS (estimate = -0.071, SE = 0.035, t = -2.022, df = 105.551, *p* = .046). In other words, the pattern of results observed in these simplified models are, in all cases, identical to the pattern observed in our initial main analysis.

## Discussion

Principal Component Analysis (PCA) of ratings of faces on abusiveness- and attractiveness-related characteristics revealed two distinct components, reflecting Perceived Abusiveness and Physical Attractiveness, respectively. This pattern of results is potentially noteworthy, since it suggests that impressions of abusiveness-related traits are not simply a consequence of the well-established attractiveness halo effect^1^. Analyses of reported dating intentions on a hypothetical dating-decisions task revealed a strong positive effect of the Physical Attractiveness component, consistent with prior work suggesting that perceptions of physical attractiveness are particularly important for dating intentions and partner choice [e.g.^2,3^].

Contrary to our predictions, the Physical Attractiveness and Perceived Abusiveness components did not interact to predict reported dating intentions. This null result for the interaction between the Physical Attractiveness and Perceived Abusiveness components is somewhat surprising, given previous work reporting that undesirable traits had weaker negative effects on reported dating intentions when assessing more attractive individuals^10,11^. Indeed, the Perceived Abusiveness component did not significantly predict reported dating intentions. Although this latter null result is somewhat surprising, it is possible that women are aware that such impressions are misleading (i.e., women are aware that perceptions of potential partners’ propensity to abuse their partners are often inaccurate) and discount these impressions accordingly. That women’s dating intentions were better predicted by physical attractiveness than threat-related perceptions is somewhat surprising, given previous work using a trust game that found women’s trusting behaviour was better predicted by the threateningness than physical attractiveness of male partners^23^. Together these results highlight potentially substantial differences in the decision-making processes that underpin responses in trust games and hypothetical dating decisions.

Our null results for predicted effects of the Perceived Abusiveness component are at odds with Shuster et al.’s^9^ finding that threat-related perceptions of potential partners’ face images had a negative effect on reported dating intentions in a hypothetical dating-decision task. Shuster et al. altered the perceived threat of face images by experimentally manipulating face-shape characteristics associated with perceived dominance. The differences between our results and those reported by Shuster et al. are puzzling. It is possible that methodological differences between Shuster et al.’s study and our study at least partly contribute to the different patterns of results. For example, while we employed 90 face images, Schuster et al. used 4, and while we used AI generated faces, Schuster et al. used images of real individuals in which threat cues were experimentally manipulated. In Schuster et al.’s work, but not our study, the face images were also accompanied by a short text bio.

A potential limitation of the study is that different participants completed the dating-decisions task and rated the traits used to construct the perceived abusiveness and physical attractiveness components. However, on this issue, it is worth noting that some researchers have argued that using the same participants to rate predictor and outcome variables in studies of face perception can inflate the correlations between these variables [e.g.^24^]. Furthermore, the Cronbach’s alphas for all traits were reasonably high, suggesting the components are likely to generalise relatively well to new groups of participants. Nonetheless, it is possible that the lower Cronbach’s alphas for the traits used to construct the perceived abusiveness component (compared to those used to construct the physical attractiveness component) may have contributed to the null results we observed for perceived abusiveness, particularly given the emotional expressions shown in the images were somewhat homogenous (i.e., generally positively valenced). These issues may be useful to explore in future research, along with other factors previously shown to influence partner and face preferences (e.g., the temporal context of the relationship for which individuals were assessed, i.e., short- versus long-term relationships^25^). Similarly, the extent to which there are differences in responses on hypothetical dating decisions and actual partner choices are poorly understood, as are the extent to which trait ratings and spontaneously generated appearance-based first impressions. Again, such differences may contribute to our null results and are issues we recommend be considered in future research. Other directions for future research, such as testing men’s dating decisions and use of longitudinal, rather than cross-sectional, designs, might also be helpful.

The only significant effect involving any of the individual difference measures we considered in our study (sensation seeking, self-perceived mate value, sociosexual orientation, and partnership status) was a significant interaction between sensation seeking and the Physical Attractiveness component. This interaction reflected women who scored higher on a measure of sensation seeking showing a weaker positive effect of the Physical Attractiveness component. This pattern of results is consistent with other research suggesting that individuals who score higher on sensation seeking tend to generally be less ‘picky’ (i.e., discriminating) when assessing potential partners [e.g.^11,26^].

The specific facial characteristics that influence perceived abusiveness are not well known. Face-shape masculinity is one candidate cue, given it is thought to be correlated with impressions of physical dominance and aggressiveness^27^. However, it is unlikely to be the characteristic that drives perceptions of abusiveness (see^28^ for evidence that methodological issues may have led researchers to overestimate the importance of shape masculinity for dominance-related perceptions). The role of other facial characteristics (e.g., facial width-to-height ratio and/or demeanour), as well as social stereotypes related to characteristics such as body size, age, and ethnicity, may also play a role.

In conclusion, our results suggest that perceptions of attractiveness- and violence-related traits can be dissociated, that impressions of men’s physical attractiveness (but not violence-related traits) are a strong predictor of women’s reported dating intentions, and that this effect of physical attractiveness is stronger among women who score low on sensation seeking. Thus, our work presents further evidence for the importance of physical attractiveness in dating and partner choices and highlight the role sensation seeking appears to play in individual differences in the effect of men’s physical attractiveness on women’s dating intentions.

## Data Availability

All data and analysis code are publicly available on the Open Science Framework (https://osf.io/ugpxs/).
